# An enigmatic new species of *Panorpa* Linneaus from the Bashan Mountains (Mecoptera, Panorpidae)

**DOI:** 10.3897/zookeys.777.26056

**Published:** 2018-07-30

**Authors:** Yuan Hua, Shi-Heng Tao, Bao-Zhen Hua

**Affiliations:** 1 College of Life Sciences, Northwest A&F University, Yangling, Shaanxi 712100, China Northwest A&F University Yangling China; 2 Key Laboratory of Plant Protection Resources and Pest Management, Ministry of Education, Entomololgical Museum, Northwest A&F University, Yangling, Shaanxi 712100, China Northwest A&F University Yangling China

**Keywords:** China, Hubei, Oriental Region, Mecoptera, *
Panorpa
*, Panorpidae, Shaanxi

## Abstract

A new species of Panorpidae, *Panorpabashanicola***sp. n.**, is described and illustrated from the Bashan Mountains in central China. The new species is characterized by the following characters: vertex black, with two pale longitudinal stripes and four pale rounded spots; vein 1A ending before the origin of Rs; meso- and metanotum pale, and the pale color extending to tergum III in V-shape; male epandrium emarginate distally in deep U-shape; hypovalves without basal stalk, completely represented by a pair of short hypovalves, extending to distal third of gonocoxite, with five black stout setae in distal portion; paramere simple, S-shaped; a bundle of long hairs between dorsal and ventral valves of aedeagus; dorsal valves of aedeagus much longer than ventral valves and curved ventrally, with distal portion foot-shaped; female medigynium twice as long as wide, with stout axis extending over one-third its length beyond main plate.

## Introduction

Panorpidae is the largest family of Mecoptera, with more than 420 described extant species, which are assigned to seven genera (Gao et al. 2016). *Panorpa* Linnaeus, 1758 is the most species-rich genus in Panorpidae, and is widely distributed in Asia, Europe and North America (Esben-Petersen 1921; Cheng 1957), with approximately 270 species in the world (Penny and Byers 1979) and 229 species in China (Wang and Hua 2017, 2018).

**Figure 1.**
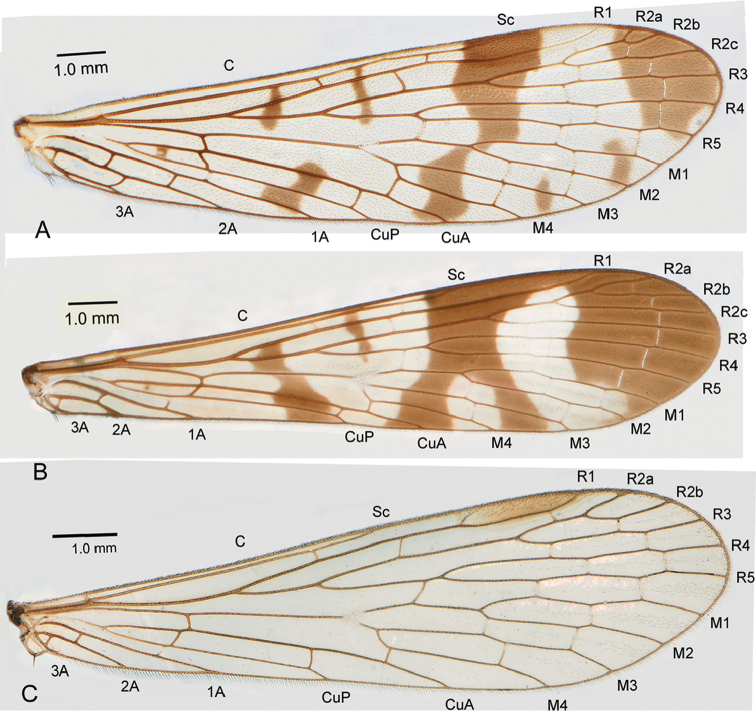
Forewings of male Panorpidae. **A***Panorpacommunis***B***Neopanorpapulchra***C***Panorpabashanicola* sp. n.

The current generic taxonomy of Panorpidae strongly relies on the status of vein 1A to a large extent as well as the presence or absence of anal horn(s) on the posterior margin of tergum VI in males, apart from the male and female genitalia (Carpenter 1938). Vein 1A ends at the anal margin of wings far beyond the origin of Rs in *Panorpa* Linneaus, 1758 (Fig. 1A), but before the origin of Rs in *Neopanorpa* van der Weele, 1909 (Fig. 1B) (Esben-Petersen 1921; Cheng 1957; Rust and Byers 1976; Chau and Byers 1978). The number of cross veins between veins 1A and 2A of forewings are also used as a generic character: two in *Panorpa*, but one in *Neopanorpa* (Esben-Petersen 1921; Chau and Byers 1978). *Panorpa* is also separated from *Neopanorpa* by a series of morphological characters (Ma et al. 2012) and anatomical characters, such as salivary glands (Ma et al. 2011), female genital plate (Ma et al. 2012), and female reproductive system (Hou and Hua 2008). Eggshell (Ma et al. 2009) and larvae (Yie 1951) can also provide useful characters.

However, some Chinese Panorpidae, such as *Panorpafulvastra* Chou and *P.chengi* Chou (Chou et al. 1981), have vein 1A ending just at the level of the origin of Rs (Cai et al. 2008), making vein 1A not so credible as a diagnosis to differentiate *Panorpa* from *Neopanorpa*.

Further complicating the issue is an enigmatic undescribed species from the Bashan Mountains in central China. Its wing venation belongs to the pattern of *Neopanorpa* with 1A ending before the origin of Rs and one cross-vein between veins 1A and 2A (Fig. 1C), while other characters, especially the male and female genitalia, correspond to the genus *Panorpa*. In this paper, we describe the new species in *Panorpa* Linneaus, 1758 mainly based on the characters of genitalia, and briefly discuss the current criteria of the generic diagnoses of *Panorpa* and *Neopanorpa*.

## Materials and methods

The specimens were collected from the Bashan Mountains in central China, and are preserved in 70% alcohol at the Entomological Museum, Northwest A&F University, China (**NWAU**). Observations were made under a Nikon SMZ1500 stereoscopic zoom microscope. Photographs were taken with a Nikon CoolPix5000 digital camera attached to the microscope.

For scanning electron microscopy, samples were cleaned in an ultrasonic cleaner for 30 s and dehydrated in a graded ethanol series. The materials were then dried in a CO_2_ critical-point drier, gold-coated in a sputter coater and examined in a Hitachi S-3400N scanning electron microscope (Hitachi, Tokyo, Japan) at 15 kV.

## Taxonomy

### Panorpa bashanicola sp. n.

Taxon classificationAnimaliaMecopteraPanorpidae

http://zoobank.org/95659BA8-69DE-4EFA-A507-565E3D14FDD4

**Figure 2.**
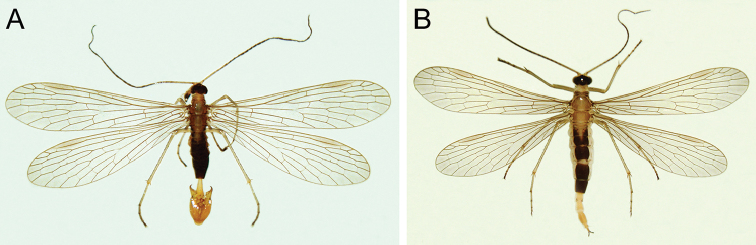
*Panorpabashanicola* sp. n., adults in dorsal view. **A** Male **B** Female.

**Figure 3.**
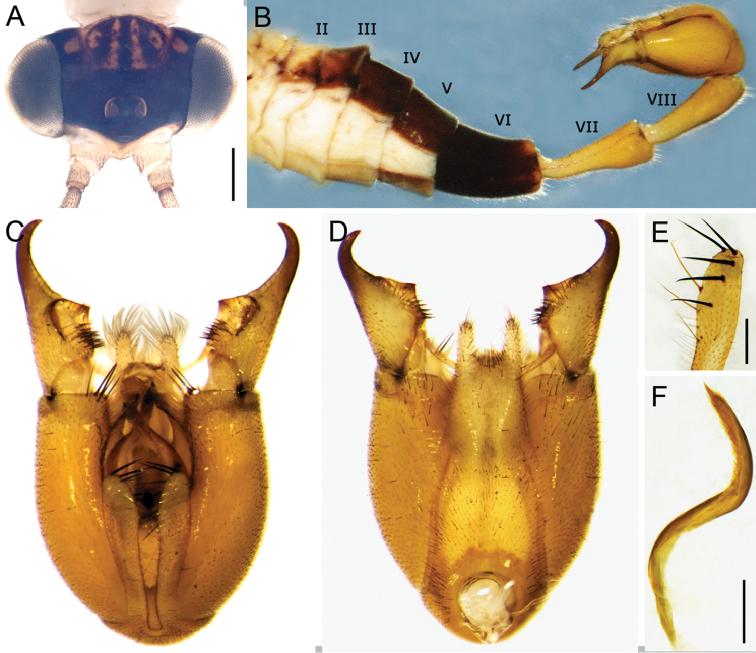
*Panorpabashanicola* sp. n., male. **A** Head in dorsal view **B** Abdomen in lateral view **C, D** Genital bulb in ventral and dorsal views **E** Distal part of left hypovalves in dorsal view **F** Left paramere in ventral view. Scale bars: 0.5 mm (**A**); 0.2 mm (**E, F**).

**Figure 4.**
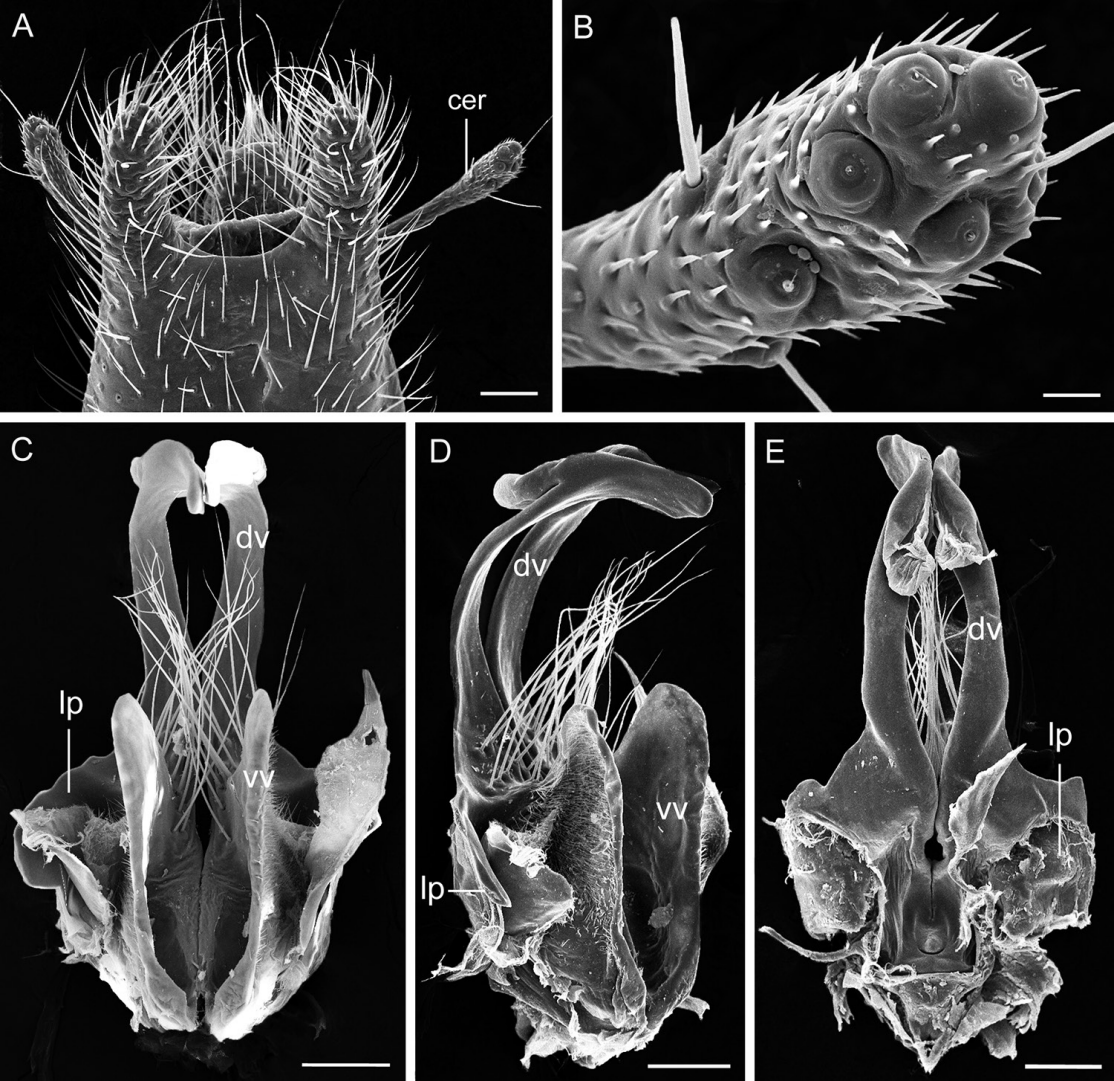
SEM micrographs of the male genitalia of *Panorpabashanicola* sp. n. **A** Distal part of epandrium in dorsal view **B** Magnification of distal part of cercus **C–E** Aedeagus in ventral, lateral and dorsal views. Abbreviations: **cer** cercus; **dv** dorsal valve of aedeagus; **lp** lateral process; **vv** ventral valve of aedeagus. Scale bars: 1 mm (**A, B**); 100 μm (**C–E**).

**Figure 5.**
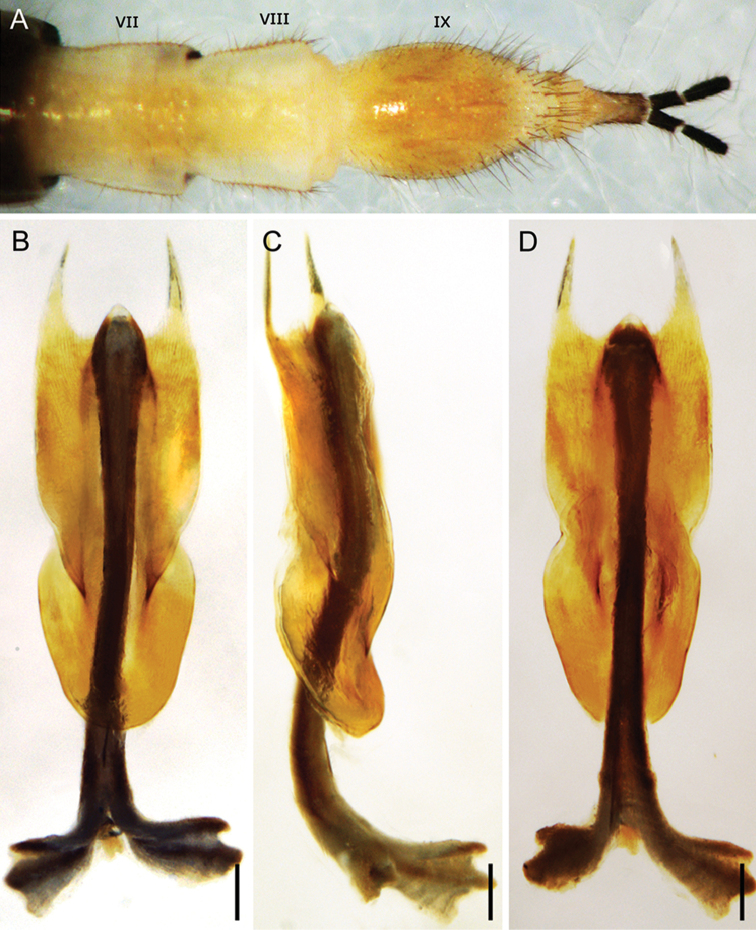
*Panorpabashanicola* sp. n., female. **A** Distal part of abdomen in ventral view **B–D** Medigynium in ventral, lateral and dorsal views. Scale bars: 0.1 mm.

**Figure 6.**
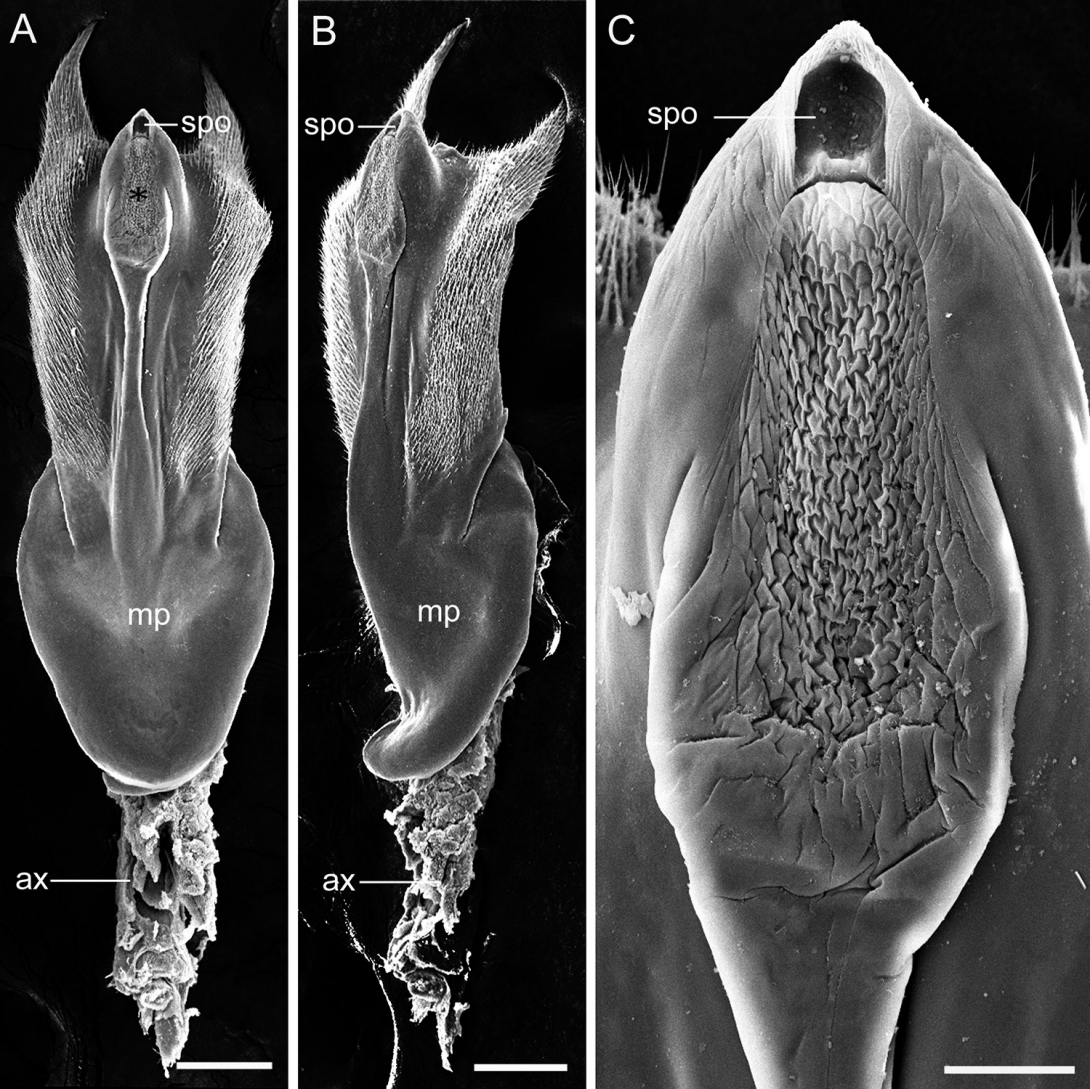
SEM micrographs of the female medigynium of *Panorpabashanicola* sp. n. **A, B** Medigynium in ventral and latero-ventral views **C** Magnification of the sculpturing part of the main plate (asterisk), showing the orifice of spermathecal duct. Abbreviations: **ax** axis; **mp** main plate; **spo** orifice of spermathecal duct. Scale bars: 100 μm (**A, B**); 25 μm (**C**).

Figs 1 , 2 , 3 , 4 , 5 , 6

#### Type material.

**Holotype**: ♂, CHINA: Shaanxi: Nangongshan (32°14'N, 109°04'E), 1200–2025 m, Langao County, 24–25 June 2007, BZ Hua and JL Tan. **Paratypes**: 26♂22♀, same data as holotype; 12♂23♀, Nangongshan, 17–18 Aug. 2010, BZ Hua, J Huang, J Chen, JX Zhang; 40♂62♀, Nangongshan, 17–19 July 2011, BZ Hua, QH Gao, M Wang, B Xu; 24♂63♀, Nangongshan, 17–18 June 2012, BZ Hua, N Ma, B Xu, QH Gao, YY Feng; 2♂, Hubei, Hongping (31°20'N, 110°22'E), Shennongjia, 28 June 2007, BZ Hua and JL Tan.

#### Diagnosis.

The new species can be readily distinguished from its congeners by the following characters: vertex black, with two pale longitudinal stripes and four pale rounded spots; vein 1A ending before the origin of Rs; one cross-vein between veins 1A and 2A; meso- and metanotum pale, and the pale color extending to tergum III in a V-shape; male epandrium emarginate distally in a deep U-shape; hypovalves extending to the distal third of gonocoxite, with five stout black setae on the distal portion; paramere simple, S-shaped; a bundle of long hairs between the dorsal and ventral valves of aedeagus; dorsal valves of aedeagus much longer than ventral valves and curved ventrally, with distal portion foot-shaped; female medigynium twice as long as wide, with stout axis extending over one-third its length beyond main plate.

#### Description of male

(Fig. 2A). Vertex black, with two pale submedian stripes and two eye-shaped speckles on lateral regions. Two suborbicular spots beyond the protuberant area laterally (Fig. 3A). Ocellar triangle black. Compound eyes dark grey. Rostrum uniformly yellowish, mandible dark brown; labial and maxillary palps yellow with distal segments dark brown. Antennae long, filiform, with 39–40 flagellomeres.

*Thorax*. Pronotum light brown, with 12 black setae along anterior margin; meso- and metanotum pale with both sides grayish yellow. Pleura light grayish yellow. Legs pale yellow, tibia with a pair of apical spurs; tarsi light yellowish brown.

*Wings*. Forewing length 13.0–13.2 mm, width 2.9–3.1 mm. Wing membrane hyaline, almost without markings. Apical band greatly reduced, only indicated by a narrow dark gray trace at apical region; pterostigma prominent. Vein R_2_ bifurcate; vein 1A ending before the origin of Rs; one cross-vein between veins 1A and 2A (Fig. 1C). Hindwings similar to forewings (Fig. 2A).

*Abdomen*. Terga I–V brownish black except for a narrowing pale V-shaped median stripe on terga I–III. Notal organ of tergum III very short, not prominent. Tergum VI without anal horns on posterior margin. Segments VII and VIII elongate and uniformly yellowish brown, with basal half slightly constricted and slightly wider toward apices (Fig. 3B).

*Male genitalia*. Genital bulb globular, yellowish brown (Fig. 3C, D). Epandrium (tergum IX) broad at base, slightly narrower toward apex, with a deep broad U-shaped emargination distally; epandrial lobes with dense setae. Cercus elongate and expanded apically (Fig. 4A), with five campaniform sensilla on dorsal surface (Fig. 4B). Hypandrium (sternum IX) without basal stalk, completely represented by a pair of parallel hypovalves, reaching two-thirds of gonocoxite (Fig. 3C). Hypovalve with five stout black setae on distal portion and three yellow setae on dorsal side (Fig. 3E). Gonocoxite bearing a cluster of black bristles on inner apex (Fig. 3C). Gonostylus broad in basal half and slender in distal half, with a large median concave area; a cluster of stout black setae basal to the concave region (Fig. 3C). Parameres distinctly twisted in S-shape and bearing short setae along inner margin on distal part (Fig. 3F). Aedeagus weakly sclerotized; dorsal valves slender and curved ventrally, with distal part foot-shaped; ventral valves greatly shortened; a bundle of long hairs between ventral and dorsal valves (Fig. 4C, D). A short broad lateral process from basal part of dorsal valves (Fig. 4C, E).

#### Description of female.

Head, thorax and abdominal segments I–V similar to those of male (Fig. 2B). Abdominal segments VII and VIII pale yellow, segment IX yellowish brown (Fig. 5A). Cerci black, two-segmented, arising from distal end of abdomen. Wing pattern similar to that of male (Fig. 2B).

*Female genitalia*. Subgenital plate broad subbasally and narrowing toward apex, with long setae on lateral distal part (Fig. 5A). Medigynium with main plate twice as long as wide and infolded medially. Paired posterior arms forming a broad U-shape emargination. Median axis stout, extending anteriorly over one-third its length beyond main plate (Fig. 5B–D), with anterior end broadly furcate. Posterior end of axis with sculptured region, with orifice of spermathecal duct at terminal end (Fig. 6A–C).

#### Etymology.

The specific epithet, *bashanicola*, refers to its type locality, Bashan Mountains.

#### Distribution.

China (Shaanxi and Hubei).

#### Remarks.

The new species resembles *P.chengi* Chou, 1981 from the Qinling Mountains, Shaanxi Province in pale nota and brownish body coloration as well as broad hypovalves. It can be readily recognized from the latter by the following characters: 1) vein 1A ending before the origin of Rs; 2) abdominal terga I–III brownish black except for a V-shaped pale median stripe; 3) parameres bearing dense setae along inner margin of distal portion; and 4) dorsal valves of aedeagus slender and foot-shaped in distal portion.

## Discussion

We assigned the new species to *Panorpa* Linneaus based on the following characters: notal organ on tergum III in male not prominent; hypandrium of male genitalia without basal stalk; female medigynium with long axis extending anteriorly beyond main plate by one third length.

However, vein 1A terminates at the hind margin of wings before the origin of Rs in the new species. Strictly speaking, this character is not in accord with the generic definition of *Panorpa* Linnaeus, but conforms to the genus *Neopanorpa* van der Weele (Esben-Petersen 1921; Chau and Byers 1978; Cheng 1957). Because vein 1A varies considerably among the species of Panorpidae, taxonomists should be cautious to assign a species to the suitable genus based mainly on the character of vein 1A. Instead, it is more reliable for them to consult more characters, especially the male and female genital characters (Carpenter 1938). In other words, the genus *Neopanorpa* needs to be redefined accordingly.

## Supplementary Material

XML Treatment for Panorpa bashanicola
